# Early erythrocyte heterogeneity in very preterm infants conceived by in vitro fertilization: a multicenter study

**DOI:** 10.1186/s12887-026-06755-0

**Published:** 2026-03-20

**Authors:** O. Serce Pehlevan, T. Donmez, B. Livaoglu Say

**Affiliations:** 1 Neonatology Unit, Kocaeli University School of Medicine, Kocaeli, Turkey; 2 Neonatology Unit, Derince Education and Training Hospital, Kocaeli, Turkey; 3 Neonatology Unit, Alaaddin Keykubat University School of Medicine, Alanya, Turkey

**Keywords:** Complete blood count, In vitro fertilization, Morbidity, Platelet indices, Prematurity, Red cell distribution width

## Abstract

**Background:**

To evaluate the association between in vitro fertilization (IVF) conception and early hematological indices reflecting erythrocyte heterogeneity and platelet characteristics in preterm infants, and to explore their relationship with morbidities.

**Methods:**

This multicenter retrospective cohort study included preterm infants (< 34 weeks’ gestation, ≤ 2000 g). Red cell distribution width (RDW), mean platelet volume (MPV), platelet distribution width, RDW-to-platelet ratio, and platelet count obtained on admission were compared between IVF- and non-IVF-conceived infants. Multivariable linear regression analyses adjusted for gestational age, maternal preeclampsia, and plurality were used to identify independent associations with hematological indices. Multivariable logistic regression analysis assessed determinants of severe composite morbidity (Bronchopulmonary dysplasia, intraventricular hemorrhage grade III-IV, necrotizing enterocolitis stage ≥ 2, treatment-requiring retinopathy of prematurity).

**Results:**

Among 453 infants, 17.7% were conceived by IVF. IVF-conceived infants had lower MPV and higher RDW in univariable analyses, while other platelet-related indices did not differ. After adjustment for gestational age, preeclampsia, and plurality, IVF conception remained independently associated with higher RDW (B = 0.95, 95% CI 0.36–1.54; *p* = 0.002). Retinopathy of prematurity and treatment-requirement were more frequent among IVF infants in unadjusted analyses, whereas severe composite morbidity was less frequent. In multivariable logistic regression, gestational age and prolonged rupture of membranes were the main determinants of morbidity; while RDW was not.

**Conclusions:**

IVF conception is independently associated with higher early RDW values in preterm infants, indicating increased erythrocyte size heterogeneity at birth. Platelet-related indices were not independently associated with mode of conception. RDW was not associated with neonatal morbidity suggesting that this hematologic difference reflects variation in early neonatal hematologic characteristics rather than a predictor of adverse outcomes.

## Introduction

In vitro fertilization (IVF) is increasingly used worldwide to treat infertility. Pregnancies conceived by this technology have been reported to exhibit subtle alterations in placental function, intrauterine oxygenation, and inflammatory balance which may influence fetal hematopoiesis [1]. Hematological indices derived from the complete blood count provide a readily available and cost-effective means of evaluating these processes in the neonatal period.

Platelets are not only central to hemostasis but also actively participate in inflammatory responses and endothelial interactions. Platelet-related indices such as mean platelet volume (MPV), platelet distribution width (PDW), and platelet-to-red cell ratio (RPR) reflect platelet activation, size heterogeneity, and platelet–erythrocyte interactions respectively, whereas the platelet count represents the quantitative aspect of thrombopoiesis [2, 3]. In parallel, red cell distribution width (RDW) reflects erythrocyte size heterogeneity and has emerged as a marker of ineffective erythropoiesis, systemic stress, and inflammatory states in neonates [4–6].

Despite growing interest in neonatal hematological markers, data examining the combined behavior of platelet indices and RDW in very preterm infants conceived via IVF remain limited. The unique biological milieu of IVF gestations suggests that these infants may exhibit distinct early hematologic patterns independent of overt clinical outcomes. However, the clinical implications of such differences remain uncertain. Therefore, this multicenter retrospective cohort study aimed to compare early hematologic indices measured after birth in very preterm infants conceived by IVF with those conceived spontaneously, and to explore their association with major neonatal morbidities.

## Methods

This multicenter retrospective cohort study used clinical data collected between 2016 and 2021 from three neonatal intensive care units (NICU) in the same metropolitan area. These units serve as major referral centers for neonatal care in the region and collectively manage a substantial proportion of preterm deliveries. The study protocol was reviewed and approved by the local ethics committee (2021/76). This study was performed in accordance with the Declaration of Helsinki. Eligible participants included neonates born at less than 34 weeks of gestation with a birth weight below 2000 g. Infants with major congenital anomalies or early culture-proven sepsis were excluded from the analysis.

The primary exposure of interest was conception by IVF. Demographic, perinatal, and clinical data were retrieved from electronic medical records. Collected variables included gestational age, birth weight, sex, plurality (singleton or multiple gestation), mode of delivery, maternal preeclampsia, placental complications, and small-for-gestational-age status. Gestational age was determined based on first-trimester ultrasonography or obstetric dating records. Hematological parameters (platelet count, MPV, PDW, RPR, and RDW) were obtained from the first complete blood count performed immediately upon NICU admission, prior to any blood transfusion or major therapeutic intervention.

The primary outcomes were the early profiles of hematologic indices after birth including PDW, RDW, and MPV. Secondary outcomes included RPR and PLT. Clinical outcomes and neonatal morbidities were not evaluated as primary endpoints and were considered only in exploratory analyses. Although clinical outcomes were not predefined primary endpoints, severe composite morbidity was included in secondary multivariable analyses to assess the potential clinical relevance of observed hematologic differences.

IVF conception was defined as pregnancy achieved through assisted reproductive techniques including in vitro fertilization with or without intracytoplasmic sperm injection. Bronchopulmonary dysplasia was defined as the requirement for supplemental oxygen at 36 weeks’ postmenstrual age. Severe necrotizing enterocolitis was defined as Bell stage ≥ II disease [7]. Retinopathy of prematurity (ROP) was diagnosed and staged according to the International Classification of Retinopathy of Prematurity [8]. ROP requiring treatment was defined as disease requiring laser photocoagulation or intravitreal anti–vascular endothelial growth factor therapy [9]. Intraventricular hemorrhage was diagnosed by cranial ultrasonography and graded according to the Papile classification [10]. Severe intraventricular hemorrhage was defined as grade III–IV hemorrhage. Severe composite morbidity was defined as the presence of at least one of the following major neonatal outcomes: bronchopulmonary dysplasia, severe intraventricular hemorrhage, severe necrotizing enterocolitis, or retinopathy of prematurity requiring treatment.

### Statistical analyses

Statistical analyses were performed using SPSS software version 25.0 (IBM Corp., Armonk, NY, USA). Continuous variables were assessed for normality using the Shapiro–Wilk test. Data are presented as medians (25 P–75 -P) for non-normally distributed variables and as means ± standard deviations for normally distributed variables. Comparisons between IVF- and non-IVF-conceived infants were performed using the Mann–Whitney U test or Student’s t test as appropriate. Categorical variables were compared using the χ² test or Fisher’s exact test.

Multivariable linear regression analyses were performed separately for MPV, RPR, and RDW to evaluate the independent association between IVF conception and hematologic indices. Based on clinical relevance and the anticipated confounding structure gestational age, maternal preeclampsia, and plurality were included as covariates in all final models. All predictors were entered simultaneously using the enter method. Additional perinatal variables (sex and small-for-gestational-age status) were initially evaluated but were not retained in the final parsimonious model, as they were not independently associated with the outcomes and did not materially alter the IVF coefficient. Regression coefficients are presented as unstandardized B values with corresponding 95% confidence intervals. Multicollinearity was assessed using variance inflation factors (VIF) with values < 2 considered acceptable.

A predefined sensitivity analysis was conducted restricting the cohort to singleton pregnancies to address potential confounding by plurality. The association between IVF conception and RDW was re-evaluated using multivariable linear regression adjusted for gestational age and maternal preeclampsia in this subset.

Multivariable logistic regression analysis was performed for severe composite neonatal morbidity to explore clinical relevance. Covariates included gestational age, sex, plurality, prolonged rupture of membranes (> 18 h), maternal preeclampsia, small-for-gestational-age status, RDW, and IVF conception. Adjusted odds ratios (OR) with 95% confidence intervals are reported. A two-sided p value < 0.05 was considered statistically significant.

## Results

### Study population and baseline characteristics

A total of 482 neonates with a gestational age < 34 weeks and birth weight ≤ 2000 g were admitted to the NICU during the study period. After exclusion of infants with major congenital anomalies (*n* = 16) and early culture-proven sepsis (*n* = 13), 453 very preterm infants were included in the final analysis. Of these, 80 (17.7%) were conceived by IVF and 373 (82.3%) were conceived spontaneously.

Infants in the IVF group had a slightly higher gestational age than those in the non-IVF group (30 ± 2 vs. 29 ± 3 weeks, *p* = 0.008), whereas birth weight was lower in the IVF group [median (25 P-75 P): 1330 (1040–1682) g vs. 1510 (1217–1728) g, *p* = 0.006)]. Male sex and plurality (multiple gestation) were more frequent among IVF-conceived infants (67.5% vs. 50.4%, *p* = 0.005; and 81.3% vs. 24.7%, *p* < 0.001, respectively). Cesarean delivery was also more common in IVF pregnancies (98% vs. 87%, *p* = 0.01). Median 5-minute Apgar scores were slightly higher in the IVF group [8 (8–8) vs. 8 (6–8), *p* = 0.035)]. Maternal preeclampsia was less frequent among IVF pregnancies (10% vs. 25.7%, *p* = 0.002). Baseline characteristics are summarized in Table 1.

**Table 1:** Perinatal characteristics of the neonates

	IVF (*n* = 80)	Non-IVF (*n* = 373 )	*p* value
Gestational age (weeks), *mean ± SD*	30 *±* 2	29 *±* 3	***0.008***
Birth weight (g), *median (25 P-75 P)*	1330 (1040–1682)	1510 (1217–1728)	***0.006***
Male, *n (%)*	54 (67.5)	190 (50.4)	***0.005***
Multiple gestation, *n (%)*	65 (81.3)	93 (24.7)	***< 0.001***
Cesarean delivery, *n (%)*	78 (98)	327 (87)	***0.010***
5- minute APGAR score, *median (25 P-75 P)*	8 (8–8)	8 (6–8)	***0.035***
Small for gestational age, *n (%)*	6 (7.5)	42 (11.1)	0.33
Maternal preeclampsia, *n (%)*	8 (10)	97 (25.7)	***0.002***
Placental complications, *n (%)*	2 (2.5)	8 (2.1)	0.83

Bold values indicate statistically significant differences (p < 0.05)

### Hematologic parameters according to IVF status

MPV (fL) was lower in IVF-conceived infants compared with spontaneously conceived infants [median (25 P–75 P): 7.6 (6.9–9.5) vs. 8.2 (7.2–9.7); *p* = 0.037]. RDW (%) was significantly higher in the IVF group [17.6 (16.7–18.5) vs. 16.4 (15.0–17.6); *p* < 0.001]. Although RPR values were numerically higher in IVF infants, the difference did not reach statistical significance [0.080 (0.069–0.096) vs. 0.076 (0.059–0.097); *p* = 0.055]. Other hematologic parameters were comparable between groups (Table 2).

**Table 2:** Comparison of hematological parameters in preterm infants conceived by in vitro fertilization (IVF) and spontaneous pregnancy

	IVF (*n* = 80)	Non-IVF (*n* = 373)	pvalue
Platelet count (x10⁹/L), *median* (25 *P*-75 *P*)	219 (185–245)	210 (172–260)	0.72
Mean platelet volume (MPV, fL), *median (25 P-75 P)*	7.6 (6.9–9.5)	8.2 (7.2–9.7)	***0.037***
Platelet distribution width (PDW, %), *median (25 P-75 P)*	17.3 (14.4–18.4)	17 (14-18.1)	0.10
Red cell distribution width (RDW, %), median (25 P-75 P)*	17.6 (16.7–18.5)	16.4 (15-17.6)	***< 0.001***
RDW-to-platelet ratio (RPR), *median (25 P-75 P)*	0.080 (0.069–0.096)	0.076 (0.059–0.097)	0.055

*MPV* mean platelet volume, *PDW* platelet distribution width, *RPR* RDW-to-platelet ratio, *RDW* red cell distribution width. * Mean + SD; 17.6 *±* 1.6 vs. 16.1 *±* 2.3

In multivariable linear regression analysis adjusted for gestational age, maternal preeclampsia, and plurality, IVF conception was independently associated with higher RDW (B = 0.95; 95% CI 0.36–1.54; *p* = 0.002). Gestational age was positively associated with RDW (B = 0.26 per week; 95% CI 0.16–0.32; *p* < 0.001), whereas maternal preeclampsia was inversely associated with RDW (B = − 0.59; 95% CI − 1.07 to − 0.11; *p* = 0.029). Plurality (Multiple gestation) was also independently associated with higher RDW (B = 0.48; 95% CI 0.01–0.96; *p* = 0.044). The model explained approximately 13% of the variance in RDW (R² = 0.13), and multicollinearity was not detected (all VIF < 1.3) (Table 3).

**Table 3:** Multivariable linear regression analysis of hematologic indices

Variable	MPV B (95% CI); *p*-value	RPR B (95% CI); *p*-value	RDW B (95% CI); pvalue
In vitro fertilization	−0.36 (− 0.77 to 0.05); 0.08	0.002 (− 0.01 to 0.01); 0.76	0.95 (0.36–1.54); 0.002
Gestational age (weeks)	−0.02 (− 0.08 to 0.04); 0.47	0.001 (− 0.001 to 0.003); 0.16	0.26 (0.16–0.32); <0.001
Preeclampsia	−0.03 (− 0.40 to 0.35); 0.88	0.007 (− 0.003 to 0.017); 0.16	−0.59 (− 1.07 - −0.11); 0.017
Multiple gestation	0.03 (− 0.34 to 0.40); 0.89	0.004 (− 0.006 to 0.014); 0.49	0.48 (0.01–0.96); 0.044

Values represent unstandardized regression coefficients (B) with 95% confidence intervals. Separate multivariable linear regression models were constructed for MPV, RPR, and RDW. All covariates were entered simultaneously using the enter method and adjusted for gestational age, maternal preeclampsia, and plurality (multiple gestation)

*MPV* mean platelet volume, *RPR* red cell distribution width–to–platelet ratio, *RDW* red cell distribution width

In a predefined sensitivity analysis restricted to singleton pregnancies (IVF *n* = 15; non-IVF *n* = 282), RDW remained significantly higher in IVF-conceived infants [median 17.7% vs. 16.3%; *p* = 0.018]. In multivariable analysis within this subset, IVF conception remained independently associated with higher RDW after adjustment for gestational age and maternal preeclampsia (B = 1.32; 95% CI 0.11–2.53; *p* = 0.033). The standardized mean difference corresponded to a moderate effect size (Hedges g = 0.53). Gestational age did not differ meaningfully between IVF and non-IVF singletons (g = − 0.15).

### Neonatal morbidities according to IVF status

Major neonatal morbidities according to mode of conception are presented in Table 4. ROP occurred more frequently among IVF-conceived infants compared with spontaneously conceived infants (47.1% vs. 34.4%, *p* = 0.048), and the need for treatment was also higher in the IVF group (12.5% vs. 5.0%, *p* = 0.042). In contrast, the overall rate of severe composite morbidity was lower among IVF infants (20% vs. 34.1%, *p* = 0.014). Rates of severe intraventricular hemorrhage (Grade III–IV) and severe necrotizing enterocolitis (Bell stage *≥* II) were similar between groups. Bronchopulmonary dysplasia at 36 weeks showed a trend toward a lower frequency in IVF infants, although this did not reach statistical significance (12.8% vs. 22.8%, *p* = 0.051).

**Table 4:** Major neonatal morbidities according to mode of conception

Outcome, *n* (%)	IVF (*n* = 80)	Non-IVF (*n* = 373)	*p* value
Bronchopulmonary dysplasia at 36 weeks, *n (%)*	10 (12.8)	78 (22.8)	0.051
Retinopathy of prematurity (ROP, any stage), *n (%)*	32 (47.1)	112 (34.4)	0.048
ROP requiring treatment, *n (%)*	8 (12.5)	15 (5.0)	0.042
Severe necrotizing enterocolitis, *n (%)*	4 (5.0)	7 (2.0)	0.12
Severe intraventricular hemorrhage, *n (%)*	4 (5.0)	16 (4.5)	0.77
Severe composite morbidity, *n (%)*	16 (20)	124 (34.1)	0.014

Severe intraventricular hemorrhage was defined as grade III–IV intraventricular hemorrhage. Severe necrotizing enterocolitis was defined as Bell stage ≥ II disease. Severe composite morbidity included the occurrence of at least one of the following outcomes: bronchopulmonary dysplasia at 36 weeks’ postmenstrual age, severe intraventricular hemorrhage, severe necrotizing enterocolitis, or retinopathy of prematurity (ROP) requiring treatment

Multivariable logistic regression analysis for severe composite morbidity is presented in Table 5. Gestational age remained the strongest determinant of outcome with increasing gestational age associated with a reduced risk of severe morbidity (adjusted OR 0.57 per week increase, *p* < 0.001). Prolonged rupture of membranes exceeding 18 h was independently associated with an increased risk of severe morbidity (adjusted OR 2.68, *p* = 0.002). RDW was not independently associated with severe morbidity. After adjustment for gestational age, sex, plurality, prolonged rupture of membranes, maternal preeclampsia, small-for-gestational-age status, and RDW, IVF conception was independently associated with a lower odd of severe morbidity (adjusted OR 0.46, 95% CI 0.21–0.99, *p* = 0.047).

**Table 5:** Multivariable logistic regression analysis for severe composite morbidity

Variable	Adjusted OR	95% CI	*p* value
Multiple gestation	1.11	0.60–2.05	0.74
RDW (per 1% increase)	1.05	0.94–1.17	0.41
Gestational age (weeks)	0.57	0.50–0.64	< 0.001
Male sex	1.52	0.91–2.54	0.11
Maternal preeclampsia	1.08	0.57–2.04	0.80
PROM > 18 h	2.68	1.43–5.03	0.002
Small for gestational age	1.21	0.50–2.92	0.67
IVF conception	0.46	0.21–0.99	0.047

Odds ratios (OR) are adjusted for all variables listed in the table. All covariates were entered simultaneously using the enter method

*RDW* Red cell distribution width, *PROM* Premature rupture of membranes, *IVF* In vitro fertilization

## Discussion

We evaluated the association between IVF conception and early neonatal hematological indices in this multicenter retrospective study. Our primary finding indicates that IVF conception is independently associated with higher RDW at birth even after accounting for major perinatal confounders. In contrast, while univariable analyses suggested lower MPV and borderline higher RPR in IVF infants, these associations did not persist in multivariable models. Importantly, the observed hematologic differences were not associated with an increased risk of severe neonatal morbidity in adjusted analyses.

To our knowledge, this is among the first studies specifically examining early neonatal hematologic indices in IVF-conceived very preterm infants. Previous studies examining IVF outcomes have primarily focused on maternal hematologic or inflammatory markers and long-term cardiometabolic and neurodevelopmental outcomes with limited attention to early neonatal hematologic adaptations [11, 12]. The independent association between IVF conception and elevated RDW may reflect differences in intrauterine conditions that influence erythropoietic regulation. IVF pregnancies have been reported to exhibit alterations in placental vascular architecture, spiral artery remodeling, and perfusion characteristics [13–15]. Chronic intrauterine hypoxia and altered placental perfusion could impose sustained erythropoietic stress during fetal life resulting in increased erythrocyte size heterogeneity at birth. Such placental differences may therefore contribute to subtle alterations in erythrocyte maturation, reflected as increased RDW at birth. In addition, epigenetic modifications associated with assisted reproductive technologies linked to altered DNA methylation patterns affecting developmental regulation have been described in placental and developmental tissues [16, 17]. Although the relevance of these findings to neonatal hematologic indices remains uncertain, differential regulation of genes involved in hematopoiesis or iron metabolism represents a biologically plausible pathway that warrants further investigation. Importantly, these proposed mechanisms should be considered hypothesis-generating. Our findings demonstrate an association rather than a causal relationship between IVF conception and RDW levels. Prospective studies incorporating placental histopathology, oxidative stress markers, and molecular profiling are required to clarify whether IVF-related intrauterine differences contribute to variations in early erythrocyte heterogeneity. Our findings suggest that routinely available neonatal hematologic indices may capture subtle systemic differences associated with IVF conception, although further mechanistic studies are needed to confirm this interpretation. In contrast, the absence of independent associations between IVF conception and platelet-related indices indicates that IVF alone is unlikely to disrupt thrombopoiesis or platelet activation pathways in very preterm infants. This erythroid-predominant pattern suggests that erythropoiesis may be more vulnerable than platelet biology to intrauterine environmental perturbations associated with IVF [18].

Although the absolute difference in RDW between groups appears numerically modest (~ 1%), its magnitude should be interpreted within the context of very preterm hematologic physiology. In our adjusted regression model, each additional week of gestational age was associated with an approximately 0.26% increase in RDW, whereas IVF conception was associated with an approximately 0.95% increase. This model-based comparison suggests that the IVF-related RDW shift is not negligible relative to a well-established determinant of hematologic maturation within the same analytical framework, although this comparison should not be interpreted as biological equivalence. Consistent with this interpretation, the standardized mean difference in the singleton sensitivity cohort corresponded to a moderate effect size (Hedges g = 0.53), indicating that the observed difference is measurable relative to the intrinsic variability of RDW in very preterm infants. Given the markedly higher prevalence of multiple gestation among IVF pregnancies, plurality represents a potential confounding factor when examining neonatal hematologic parameters. In the present study, this was addressed both by adjusting for plurality in the multivariable models and by performing a sensitivity analysis restricted to singleton pregnancies. Importantly, the association between IVF conception and higher RDW persisted in singleton-only analyses, while gestational age did not differ meaningfully between groups within this subset. Together, these findings support that the observed RDW difference is unlikely to be explained solely by plurality or gestational age imbalance, although residual confounding cannot be completely excluded.

Previous reports have suggested that very preterm infants conceived through IVF may experience higher rates of neonatal morbidities including ROP, bronchopulmonary dysplasia, and other perinatal complications [19–21]. However, these associations are frequently attenuated after adjustment for gestational age, plurality, and maternal characteristics. Consistent with these observations, IVF infants in our cohort demonstrated higher rates of ROP and treatment-requiring ROP, while severe composite morbidity was paradoxically less frequent in unadjusted analyses. This finding should be interpreted cautiously given the observational design and the potential influence of residual confounding. Overall, these findings suggest that apparent differences in neonatal outcomes are more likely related to underlying perinatal characteristics rather than a direct effect of IVF conception itself. Selection-related mechanisms, including differences in maternal characteristics or survival patterns among extremely preterm infants, may partly contribute to this pattern and warrant further investigation.Taken together, our findings expand the current understanding of hematologic characteristics in very preterm infants conceived through IVF. While most previous research has focused primarily on obstetric complications and long-term developmental outcomes, our results suggest that subtle differences in early hematologic parameters may also be present at birth. However, these differences appear to reflect variations in perinatal conditions rather than direct hematologic consequences of IVF conception. Further studies integrating placental biology, fetal oxygenation markers, and longitudinal hematologic profiling are needed to clarify the biological and clinical significance of these observations.

The strengths of this study include its multicenter design and the relatively large cohort of very preterm infants allowing evaluation of hematologic indices in a clinically relevant population. In addition, hematologic parameters were obtained from the first complete blood count performed at NICU admission reflecting routine clinical practice and minimizing potential treatment-related influences. The analysis also incorporated adjustment for key perinatal confounders and a sensitivity analysis restricted to singleton pregnancies which strengthens the robustness of the observed association between IVF conception and RDW. Several limitations should be acknowledged. First, the retrospective design precludes causal inference and remains susceptible to residual confounding despite multivariable adjustment. Second, detailed placental data and biomarkers related to oxidative stress or inflammation were not available, limiting mechanistic interpretation. Finally, information regarding underlying infertility etiologies and parental characteristics was not consistently available in the clinical records. Therefore, IVF conception in our analysis may represent a proxy for broader infertility-related biological factors rather than a direct causal exposure.

## Conclusions

IVF conception was associated with higher early RDW values in very preterm infants indicating increased erythrocyte size heterogeneity at birth. In contrast, platelet-related indices were not independently associated with mode of conception. Furthermore, IVF was not linked to an increased risk of severe neonatal morbidity after adjustment for perinatal confounders. These findings suggest that RDW may reflect differences in early neonatal hematologic characteristics rather than serving as a mediator of adverse outcomes. Further prospective studies integrating longitudinal hematologic assessment with placental and molecular data are required to clarify the biological basis and potential clinical implications of these observations.

## Data Availability

The datasets generated and/or analysed during the current study are not publicly available due to patient privacy and institutional restrictions but are available from the corresponding author on reasonable request.

## Abbreviations

OR: Odds ratio
BPD: Bronchopulmonary dysplasia
CI: Confidence interval
IVF: In vitro fertilization
IVH: Intraventricular hemorrhage
MPV: Mean platelet volume
NEC: Necrotizing enterocolitis
NICU: Neonatal intensive care unit
PDW: Platelet distribution width
PLT: Platelet count
PROM: Prolonged rupture of membranes
RDW: Red cell distribution width
ROP: Retinopathy of prematurity
RPR: Red cell distribution width-to-platelet ratio
SGA: Small-for-gestational-age
